# A comparative genomic approach to identify determinants of meropenem resistance in *Klebsiella pneumoniae* using pan-genome-wide association analysis

**DOI:** 10.3389/fmicb.2026.1851170

**Published:** 2026-06-19

**Authors:** Santhiya Vijayakumar, Sudha Ramaiah

**Affiliations:** Department of Bio-Sciences, School of Biosciences and Technology (SBST), Vellore Institute of Technology (VIT), Vellore, Tamil Nadu, India

**Keywords:** *bla* *_NDM_*, GWAS, *Klebsiella pneumoniae*, meropenem, OmpK35, β-lactamases

## Abstract

Carbapenem-resistant *Klebsiella pneumoniae* (CRKP) is a serious global health threat associated with both nosocomial and community-acquired infections. The reduced efficacy of meropenem due to diverse resistance mechanisms emphasizes the need to investigate genetic determinants and their association with meropenem susceptibility, which is explored in this study. We have performed pan-genome analysis and genome-wide association studies on 350 *K. pneumoniae* genomes with corresponding antimicrobial susceptibility data to elucidate the genetic basis of meropenem resistance. A high prevalence of sequence types, such as ST101, ST11, ST147, and ST383, was observed among meropenem-resistant genomes. KL17 and KL64 were the predominant capsular types associated with resistance phenotypes. Class-A β-lactamases were widely distributed across both resistant and susceptible genomes. Carbapenemases, including NDM and KPC variants, were predominantly detected in meropenem-resistant genomes. The pan-genome exhibited an open structure, with mobilome (12.49%) and defence-related genes (7.53%) predominant in the accessory genome. Regarding alterations in outer membrane porins, over half of the resistant genomes showed predicted truncations in OmpK35 (56.13%). Additionally, OmpK36 in resistant genomes exhibited GD, TD, SD, and D amino acid insertions that were absent in susceptible genomes. Genome-wide association analyses identified several genes significantly associated with meropenem-resistance, including *bla_NDM-1_*, *ble*, *trpF*, *cutA*, *groL*, and *groS*, along with *bla_OXA_* and multiple transposases. Overall, this study provides a comprehensive genomic framework for understanding meropenem resistance in *K. pneumoniae*, highlighting the interplay between carbapenemase production and porin modifications. These findings emphasize the necessity of ongoing genomic surveillance and improvement in effective therapeutic strategies to combat multidrug-resistant infections.

## 1 Introduction

*Klebsiella pneumoniae* (*K. pneumoniae*), an opportunistic pathogen belonging to a member of the *Enterobacteriaceae* family, accounts for nearly one-third of all infections attributed to Gram-negative pathogens. The major clinical manifestation of *K. pneumoniae* encompasses pneumonia, urinary tract infections, and sepsis (Sugumar et al., 2014; Venkatesan et al., 2026). In the past few decades, treating the *K. pneumoniae* infections has become complicated owing to the rapid emergence and widespread dissemination of multidrug-resistant strains (Rocha et al., 2022; Basu et al., 2024). The global mortality burden associated with antimicrobial resistance (AMR) is attributed to over 7,00,000 deaths annually, and without the implementation of effective interventions, this might rise to 10 million by 2025 (Raza et al., 2021). Considering this growing threat, the World Health Organization (WHO) has categorized the carbapenem-resistant *K. pneumoniae* (CRKP) as a pathogen of critical-priority (Pang et al., 2025).

Meropenem is a broad-spectrum parenteral carbapenem antibiotic used for the treatment of severe infections, particularly in critically ill patients (Baldwin et al., 2008). Its bacterial efficacy is primarily mediated through interacting with penicillin-binding proteins, which thereby disrupts the peptidoglycan biosynthesis, resulting in bacterial cell death (Cottagnoud, 2002; Dabhi et al., 2024). Although meropenem exhibits inherent stability against serine β-lactamases, particularly extended spectrum β-lactamases (ESBLs), its clinical effectiveness is largely compromised by the rapid emergence of meropenem-resistant bacterial strains due to extensive and frequent inappropriate use (Miryala et al., 2020; Rivera-Espinar et al., 2020; Joshi et al., 2026). This resistance is primarily driven by the production of carbapenemases, particularly metallo-β-lactamases (MBLs), which enzymatically hydrolyze the β-lactam ring (Malathi et al., 2019; Aurilio et al., 2022). Beyond enzymatic inactivation, other mechanisms include outer membrane porins alterations and increased expression of efflux pumps that further reduce the intracellular antibiotic accumulation and therapeutic efficacy (Vijayakumar et al., 2024; Biswas and Anbarasu, 2025).

The advent of technologies, such as next-generation sequencing, has greatly transformed the characterization of large-scale high-quality bacterial genomes, facilitating the identification of genetic determinants driving AMR, particularly against carbapenems. This study investigates the genetic basis underlying meropenem resistance across a diverse collection of *K. pneumoniae* genomes (n = 350) retrieved from a public database. The resistome and the virulome profiles were compared to identify key differences between the meropenem-resistant and -susceptible genomes. Furthermore, genomic variants were characterized, and phylogenetic relationships were inferred. Pan-genome analysis was conducted to define gene clusters within the core and the accessory genome. *Genome*-wide association study (GWAS) was conducted to assess statistically significant associations of genes, SNPs, and unitigs with meropenem phenotypes. These findings provide comprehensive insights into the genetic distinctions between meropenem-susceptible and meropenem-resistant genomes, thereby informing the clinical paradigm for therapeutic management and improving antimicrobial stewardship interventions.

## 2 Materials and methods

### 2.1 Genome retrieval

The sequence metadata for *K. pneumoniae* genomes was retrieved from the NCBI Pathogen Detection Database (accessed on 8 May 2025). *K. pneumoniae* genomes that were associated with clinical human sources with available antimicrobial susceptibility data resulted in 1,334 genomes. Of these, genomes with available meropenem susceptibility testing data, collected after 2010, and with corresponding assembled genomes were further considered. The genome assemblies were downloaded using NCBI datasets v18.9.0, resulting in a final dataset of 369 genomes (O’Leary et al., 2024). The corresponding minimum inhibitory concentration (MIC) values of meropenem for these genomes, reported in BioSample records, were interpreted according to the breakpoints defined by the Clinical & Laboratory Standards Institute (CLSI) and the European Committee on Antimicrobial Susceptibility Testing (EUCAST). The accession numbers of all genomes, along with their associated sample metadata, are provided in Supplementary file 1 Table S1A.

### 2.2 Genome quality assessment

The quality metrics, such as total assembly length, N50, GC percentage, and total number of contigs of the assembly files, were checked using QUAST v5.3.0 (Gurevich et al., 2013), and the completeness of the assemblies was evaluated using BUSCO v5.8.2 utilizing the *enterobacteriaceae*_odb12 database (Simão et al., 2015; Manni et al., 2021). Genome assemblies with more than 200 contigs and those containing ambiguous bases (Ns per 100 kbp > 0) were excluded to ensure high-quality draft genomes. After quality filtering, a final dataset of 350 genomes was retained, comprising 155 meropenem-resistant and 195 meropenem-susceptible genomes for further analysis. The average nucleotide identity (ANI) between all the genomes was calculated using ANIclustermap v2.0.1.

### 2.3 Sequence typing and capsule profiling

The sequence types (ST) of all the genomes were classified using the Pasteur scheme from the MLST module of Kleborate v3.1.3, which relies on the allelic patterns of housekeeping genes, namely *mdh*, *gapA*, *pgi*, *tonB infB*, *phoE*, and *rpoB* (Lam et al., 2021). The minimum spanning tree was visualized employing the MSTree v2 method in Grapetree v1.5.0 (Zhou et al., 2018). Capsular (K) and outer lipopolysaccharide (O) loci were identified using the Kaptive tool within Kleborate v3.1.3 (Wyres et al., 2016).

### 2.4 Genome annotation and functional characterization

The gene prediction was carried out for all the genomes using Prokka v1.14.6 (Seemann, 2014). Genes for which the matches were not found in the database were classified as “hypothetical protein” using Prokka. Functional annotation of protein sequences across all genomes was performed using COGClassifier v2.0.0 to assign them to their respective clusters of orthologous groups (COGs).

### 2.5 Antimicrobial resistance genes and virulence genes profiling

The ARGs were detected across the genomes using ABRicate v1.0.1 employing ResFinder as the reference database (Zankari et al., 2012). Virulence genes were screened against the VFDB database within ABRicate v1.0.1 (Chen et al., 2016). Detection of ARGs and virulence genes was also performed using Kleborate v3.1.3 (Lam et al., 2021). The beta-lactamase database (BLDB) was used to determine the classes of β-lactamases (Naas et al., 2017).

### 2.6 Pan-genome analysis

The pan-genome of the *K. pneumoniae* strains was characterized using Panaroo v1.5.2 (Tonkin-Hill et al., 2020) and Roary v3.13.0 (Page et al., 2015). Roary was run with a 90% sequence identity cutoff to ensure robust clustering of genes. A phylogenetic tree was inferred using IQ-tree v3.0.1 based on the alignment of the core genome (Nguyen et al., 2015). The annotation and visualization of the phylogenetic tree were carried out using Interactive Tree of Life (iTOL v7; Letunic and Bork, 2021). The gene presence–absence matrix and other plots were generated using scripts from Roary v3.13.0.

The pan-genome structure of *K. pneumoniae* was characterized using Pan-Genome Profile Analyze Tool (PanGP v1.0.1) by providing the gene presence-absence matrix generated from Panaroo output (Zhao et al., 2014). A distance-guided sampling algorithm was employed to investigate pan-genome dynamics. The open or closed nature of the pan-genome was assessed using the curve-fitting function implemented in PanGP, which applies a model derived from Heaps’ law to describe gene accumulation patterns. Functional categories were assigned to the core and accessory gene clusters obtained from the pan-genome reference produced by Roary using COGClassifier v2.0.0.

### 2.7 Mutation and phylogenetic analysis

The full-length protein sequences of OmpK35 (CAA09665.1) and OmpK36 (ADG56549.1) were used as queries against the proteome of the study dataset for evaluating the protein sequence similarity (Altschul et al., 1990). The alignment coverage and sequence similarity were assessed to evaluate the structural integrity of the OmpK35 and OmpK36 proteins.

Further, the genomic variants were identified using Snippy v4.6.0 against the reference genome of *K. pneumoniae* HS11286 (NC_016845.1). Core genome SNP alignments were used to compute pairwise SNP distances using SNP-dists v0.8.2. The putative recombination sites that exhibit polymorphic sites were identified and masked from the core genomic SNP-based alignment with Gubbins v3.4 (Croucher et al., 2015; Mazzamurro et al., 2024). Following recombination removal, the SNP sites were identified through snp-sites v2.5.1 (Page et al., 2016), and a maximum likelihood SNP-based tree was inferred using FastTree v2.1.11 (Price et al., 2009).

### 2.8 Genome-wide association study

GWAS was carried out to determine the association of COGs, SNPs, and unitigs with meropenem susceptibility using a fixed effect model in Pyseer v1.4.0 (Lees et al., 2018). The meropenem susceptibility data represented in binary format as resistant and susceptible were used as the phenotype profile, while the presence–absence matrix of genes from the Panaroo was used as the genetic variants. For SNP-based GWAS, variant calling files (VCFs) from all genomes were merged and normalized using bcftools v1.15.1 prior to analysis. For unitig-based GWAS, unitigs were counted using Unitigs-caller v1.3.0 (Holley and Melsted, 2020). The COGs, SNPs, and unitigs present in less than 5% genomes were excluded using the minor allele frequency flag in Pyseer v1.4.0. The population structure correction was done by using eigenvalues of the MDS decomposition of the distance matrix derived from a recombination-free phylogenetic tree (Van Wonterghem et al., 2022). The eigenvalues of the MDS model were plotted, and the MDS scaling of the pairwise distances of seven components, as the knee points, was used to correct the population structure (Malekian et al., 2021). The significance threshold for Bonferroni correction was calculated using the number of unique patterns for COGs, SNPs, and unitigs using count_patterns.py, which resulted in 1.74E-05 for COGs, 7.40E-07 for SNPs, and 8.41E-08 for unitigs. The significant unitigs were mapped to the reference *K. pneumoniae* genome and other representative genome assemblies to determine the corresponding genes they belong to (Yebra et al., 2024).

The geneotype–phenotype association across the pan-genome was also identified through Scoary v.1.6.16 by providing a gene presence–absence matrix (genotype) and meropenem phenotype as an input. The association of gene with meropenem phenotype was derived using Fisher’s exact test, with subsequent correction applied for multiple comparisons by the Benjamini–Hochberg method. Pairwise comparisons were applied to control spurious associations based on population structure using the tree generated internally by Scoary. Further, a permutation test was performed with 10,000 permutations, which does the label switching and provides an empirical *p*-value. The association of the gene was considered significant only when the Benjamini–Hochberg corrected *p*-value was < 0.05, the best and worst pairwise comparison *p*-values were < 0.05, the empirical *p*-value was < 0.05, and the odds ratio was > 1.

## 3 Results

### 3.1 Genome dataset

Among a total of 350 genomes of *K. pneumoniae* retrieved, 155 genomes were meropenem-resistant and 195 were meropenem-susceptible genomes. The collection year of all the genomes ranged between 2010 and 2024, from various isolation sources, majorly urine (33.43%) and blood (30%). The genomes belonged to various regions, including the USA: Boston (*n* = 160), Tunisia: Sfax (*n* = 81), Pakistan (*n* = 31), and others with less frequency. The classification of genomes as meropenem-resistant or meropenem-susceptible was based on the breakpoint criteria provided in the NCBI Pathogen Detection Database and is provided in Supplementary file 1 Table S1B.

### 3.2 Quality assessment

**Figure 1:**
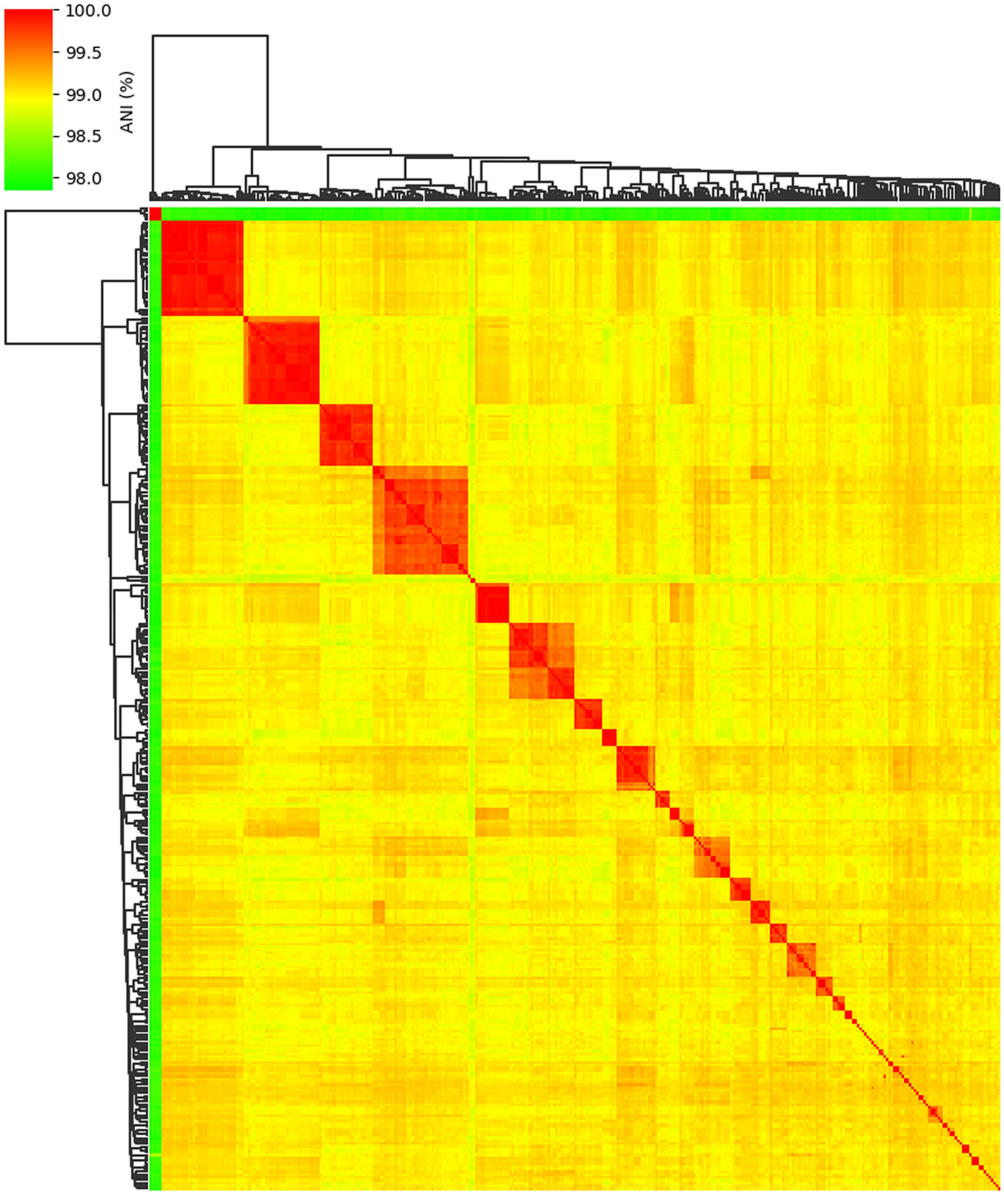
Heatmap representation of the average nucleotide identity of all *Klebsiella pneumoniae* strains. The colors represent the percentage of ANI.

The retained genomes had an average total length of ~5.70 Mbp (range: 5.14–6.27 Mbp), and the average GC content was 57.09% (range: 56.39–57.64%). The average N50 value of the genomes was 5,55,108 bp (range: 67,937–56,14,035 bp). The average number of contigs was 82 (range: 2–198; Supplementary file 2 Table S2A). The average completeness of the genomes was 98.97% (range: 97.1–99.1%; Supplementary file 2 Table S2B). The genomic similarity of each genome was determined by pairwise ANI, which was within a range of 97.84 and 100% (Figure 1).

### 3.3 Sequence typing and capsular profiling

#### 3.3.1 STs

**Figure 2:**
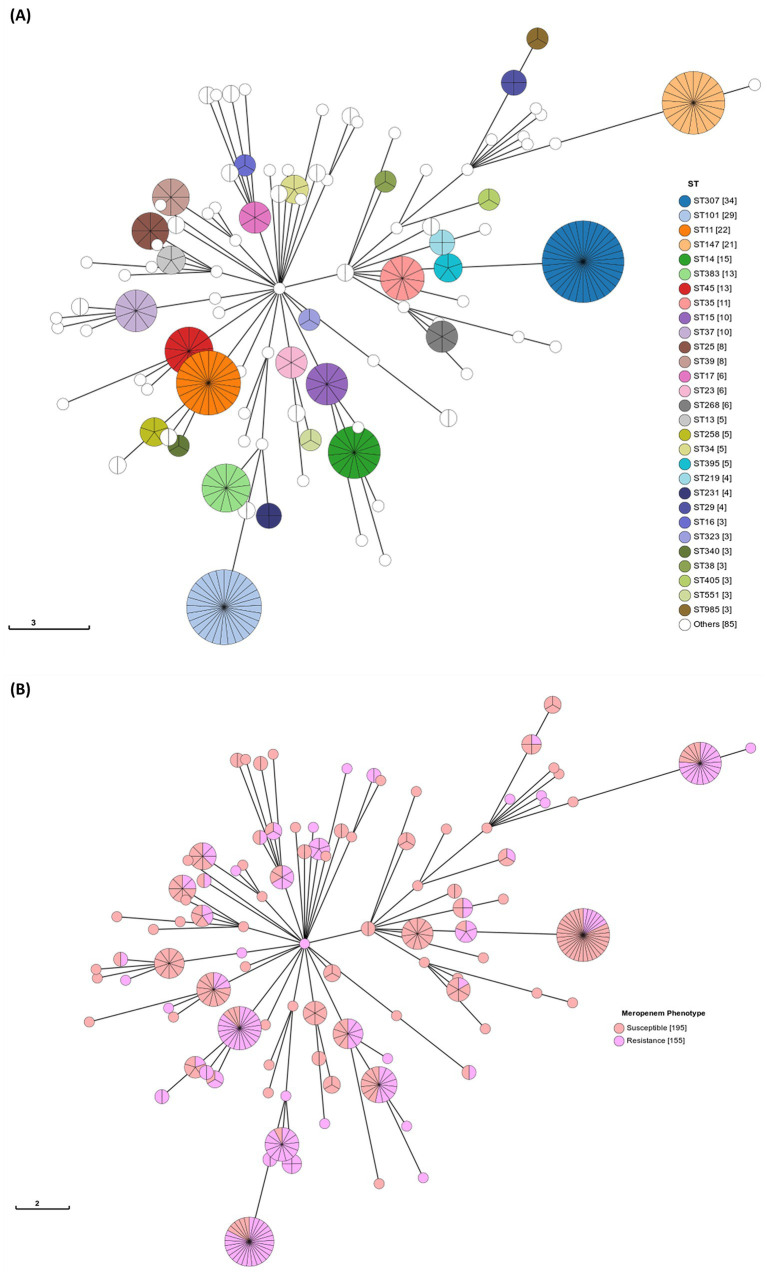
Minimum-spanning tree based on cgMLST profiles with **(A)** ST and **(B)** meropenem phenotype annotation.

There was a total of 99 STs identified among the studied genomes, and the high number of STs represents the diversity of *K. pneumoniae* in clinical settings. About 45% of the genomes accounted for STs, such as ST307, ST101, ST11, ST147, ST14, ST383, ST45, and ST35. The highest number of STs found distributed in the genome was ST307 (*n* = 34; R = 6; S = 28), ST101 (*n* = 29; R = 24; S = 5), ST11 (*n* = 22; R = 19; S = 3), ST147 (*n* = 21; R = 16; S = 5), ST14 (*n* = 15; R = 8; S = 7), ST383 (*n* = 13; R = 12; S = 1), ST45 (*n* = 13; R = 3; S = 10), and ST35 (*n* = 11; R = 0; S = 11; Table 1). There were eight loci variants for STs, such as ST147-1LV, ST258-1LV (*n* = 2), ST313-1LV, ST3164-1LV, ST603-1LV, ST666-2LV, and ST87-1LV (Supplementary file 2 Table S2C). The minimum-spanning tree constructed from cgMLST revealed strains clustering primarily according to their STs, while meropenem phenotypes were distributed across multiple clusters (Figures 2A,B).

**Table 1:** Distribution of top 10 STs among resistant and susceptible *Klebsiella pneumoniae* genomes.

ST	Total (n)	Resistance n (%)	Susceptible n (%)
ST307	34	6 (17.65)	28 (82.35)
ST101	29	24 (82.76)	5 (17.24)
ST11	22	19 (86.36)	3 (13.64)
ST147	21	16 (76.19)	5 (23.81)
ST14	15	8 (53.33)	7 (46.67)
ST383	13	12 (92.31)	1 (7.69)
ST45	13	3 (23.08)	10 (76.92)
ST35	11	0 (0.00)	11 (100)
ST15	10	5 (50)	5 (50)
ST37	10	0 (0.00)	10 (100)

#### 3.3.2 Capsular profiling

There were, in total, 63 different types of capsular types present among the genomes. The highest number of capsular types were KL102 (*n* = 36; R = 7; S = 29), KL17 (*n* = 29; R = 22; S = 7), KL64 (*n* = 26; R = 21; S = 5), KL2 (*n* = 25; R = 12; S = 13), KL24 (*n* = 23; R = 12; S = 11), KL30 (*n* = 18; R = 14; S = 4), and KL15 (*n* = 14; R = 6; S = 8; Supplementary file 3 Figure S1A). There were in total 8 different O-loci present among the genomes. The highest number of O-loci present in the genomes was OL2α.1 (*n* = 147; R = 78; S = 69), followed by OL2α.2 (*n* = 125; R = 49; S = 76), OL3γ (*n* = 29; R = 8; S = 21), OL4 (*n* = 23; R = 10; S = 13), OL13 (*n* = 14; R = 6; S = 8), OL5 (*n* = 6; R = 3; S = 3), OL3α/*β* (*n* = 5; R = 1; S = 4), and OL12 (*n* = 1; R = 0; S = 1; Supplementary file 3 Figure S1B; Supplementary file 2 Table S2C).

### 3.4 Gene prediction and functional annotation

The average number of coding sequences present in the genomes was 5,338 (range: 4,771–6,019). The average number of genes present in the genomes was 5,558 (range: 4,981–6,265). Detailed information on the distribution of rRNA, tRNA, and tmRNA genes is provided in the Supplementary File 4 Table S3A. COG-based functional classification indicated that the majority of annotated sequences were assigned to metabolism-related functions (48.92%), followed by cellular processing and signaling (23.42%), information storage and processing (19.93%), and poorly characterized (7.73%; Supplementary file 3 Figure S2A).

Across the 350 genomes analyzed, the average number of predicted sequences per genome was 5,338, with values ranging from 4,771 to 6,019. Of these, an average of 4,584 sequences per genome was assigned to COG functional categories (range: 4,529–4,919), with approximately 86% of sequences being functionally classified into COG categories (range: 80.48–89.48%; Supplementary file 4 Table S3B).

Most of the genes were categorized for the functional subsystems, such as carbohydrate transport and metabolism (COG G), amino acid transport and metabolism (COG E), transcription (COG K), cell wall/membrane/envelope biogenesis (COG M), and inorganic ion transport and metabolism (COG P) in both meropenem susceptible and resistant genomes. All meropenem-resistant and -susceptible genomes carried genes associated with mobilome: prophages, transposons (COG X, 1.91% vs. 1.52%), replication, recombination and repair (COG L, 3.82% vs. 3.66%), and defense mechanisms (COG V, 2.86% vs. 2.77%; Supplementary file 3 Figure S2B). However, meropenem-resistant genomes showed higher average gene counts in these categories, with 26.2, 4.9, and 3.6% increases in COG X, COG L, and COG V, respectively, compared to meropenem susceptible genomes. The functional annotation of all the genomes is provided in Supplementary file 4 Table S3C.

### 3.5 Distribution of ARGs and virulence genes

#### 3.5.1 ARG distribution

There was a total of 2,505 unique ARGs identified in 155 meropenem-resistant genomes, ranging from 5 to 31 ARGs per genome, while 195 meropenem-susceptible genomes harbored 2,379 ARGs, ranging from 4 to 22 ARGs per genome.

**Figure 3:**
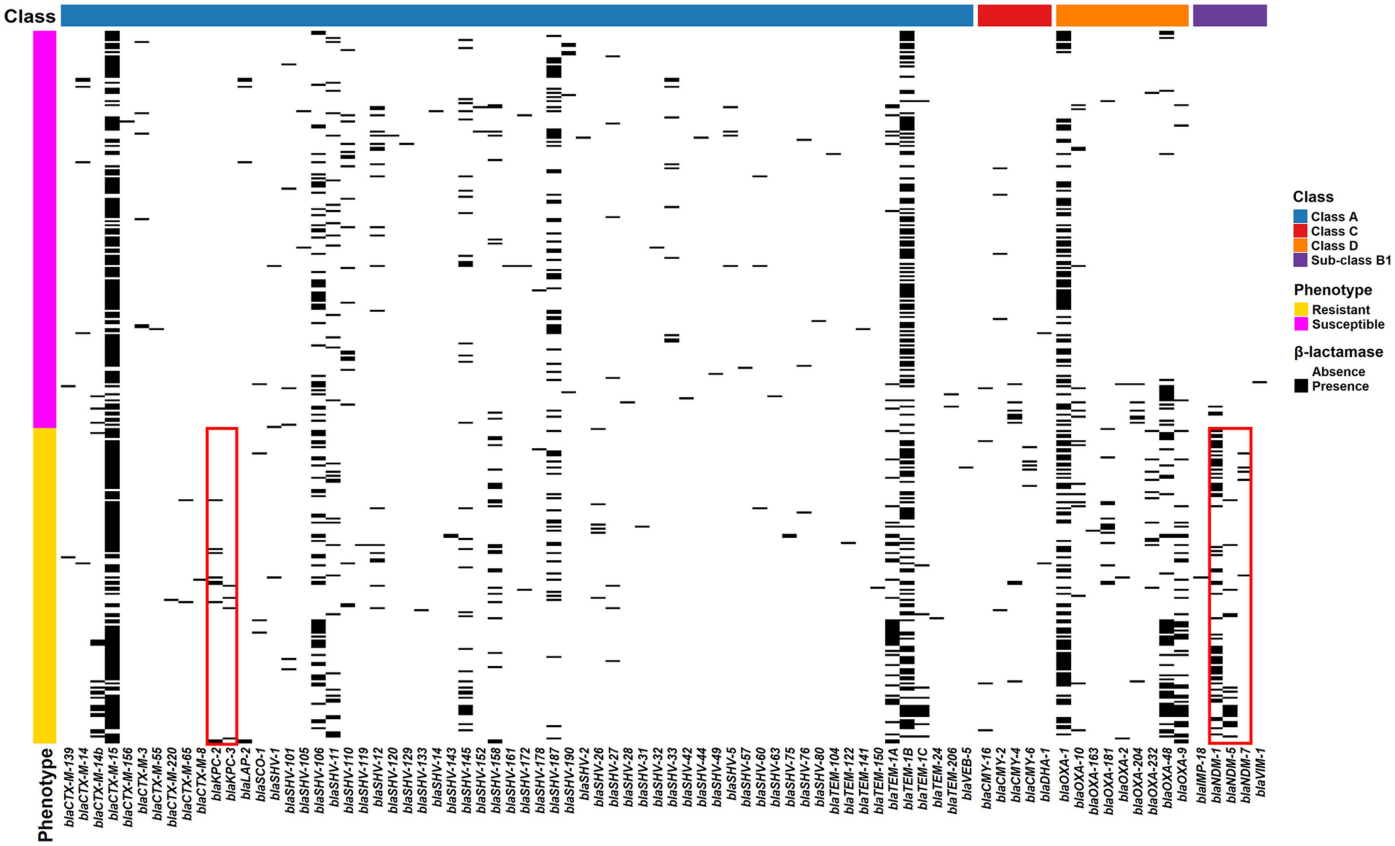
Heatmap representing the distribution of β-lactamase genes across meropenem-susceptible and -resistant *K. pneumoniae* genomes. The red-highlighted box indicates the enriched *bla_KPC_* and *bla_NDM_* genes in meropenem-resistant genomes.

β-lactamases constituted 30.78% (*n* = 771) of ARGs in resistant genomes and 26.94% (*n* = 641) in susceptible genomes. Class-A β-lactamases were predominant in both resistant and susceptible genomes, accounting for 61.35 and 78.63% of total β-lactamases, respectively. SHV β-lactamases showed high diversity with 20 variants in resistant and 32 variants in susceptible genomes. β-Lactamases, mainly *bla_NDM-1_* (*n* = 57, 36.77%), *bla_NDM-5_* (*n* = 18, 11.61%)_,_ and *bla_NDM-7_* (*n* = 5, 3.23%), belonging to class B, were high in meropenem-resistant genomes, while only 1.54% of susceptible genomes carried *bla_NDM-1_* and none of the susceptible genomes carried *bla_NDM-5_* and *bla_NDM-7_*. Similarly, OXA-type β-lactamases belonging to class D were also comparatively higher in resistant genomes. The distribution of different classes of β-lactamases across meropenem-resistant and susceptible *K. pneumoniae* genomes is represented in Figure 3. The majority of the genomes carried multiple β-lactamases, with 94.84% of meropenem-resistant genomes carrying three or more β-lactamases compared to 70.77% of susceptible genomes. Distribution of β-lactamases per genome for meropenem-resistant and susceptible *K. pneumoniae* genomes is provided in Supplementary file 3 Figure S3.

Among the meropenem-resistant genomes, *bla_NDM-1_* was the most prevalent carbapenemase identified (*n* = 44), followed by *bla_OXA-48_* (*n* = 26). Co-occurrence of multiple carbapenemases was also observed with *bla_NDM-5_* and *bla_OXA-48_* (*n* = 11), and *bla_NDM-1_* and *bla_OXA-48_* (*n* = 9) being the most common combinations. Notably, 21 meropenem-resistant genomes did not harbor any known carbapenemases. The distribution of carbapenemases and their co-occurrence patterns among meropenem-resistant genomes is shown in Supplementary file 3 Figure S4.

**Figure 4:**
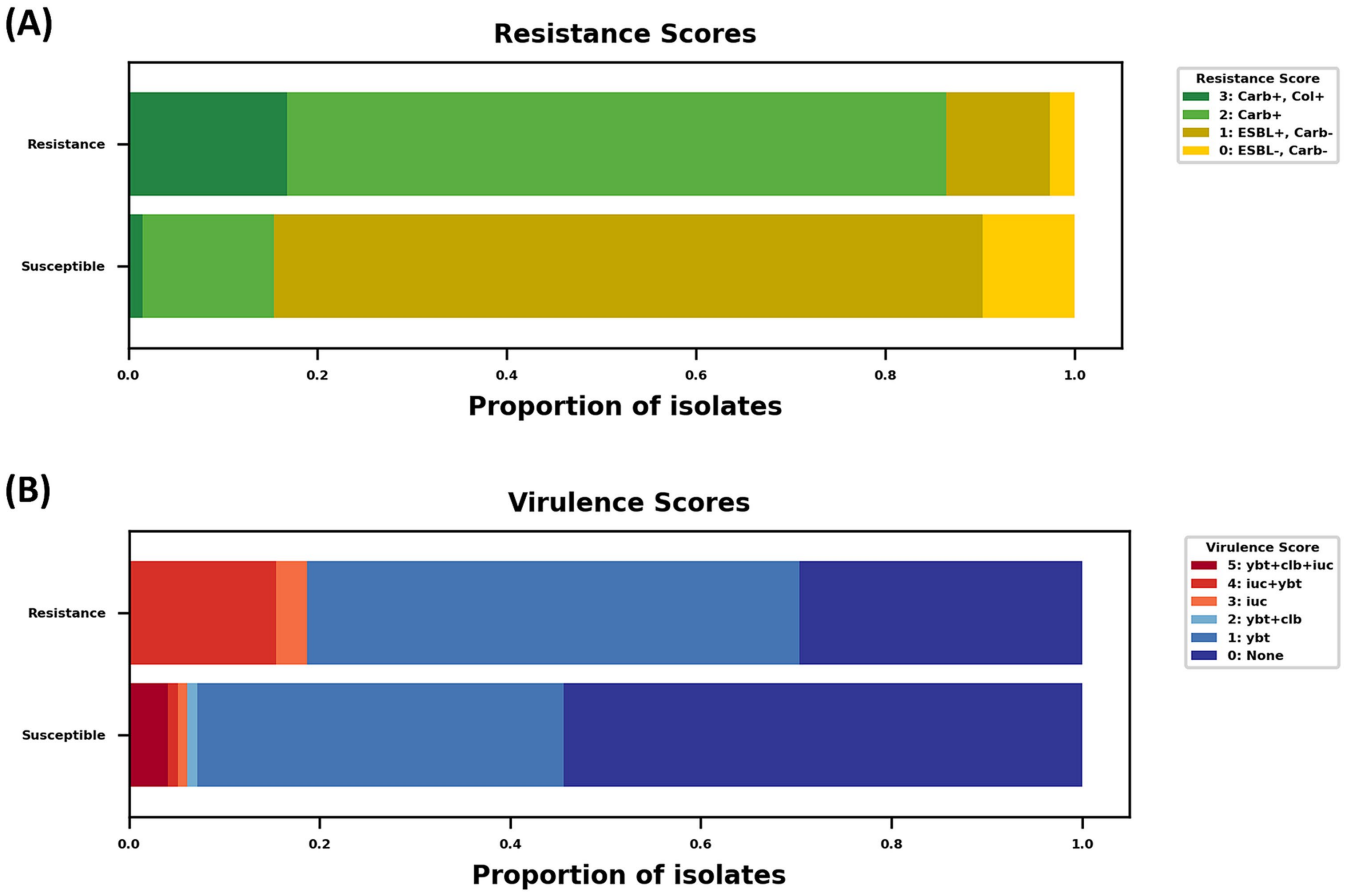
Distribution of **(A)** resistance scores based on the presence of ESBL, carbapenemase, and colistin-resistant genes and **(B)** virulence scores based on the presence of virulence gene clusters, including yersiniabactin, aerobactin, and colibactin.

Kleborate analysis further showed that carbapenemases alone were most common in resistant genomes (69.68%; score 2), followed by co-harboring carbapenemases and colistin resistance determinants (16.77%; score 3), whereas these were less frequent in susceptible genomes (13.85 and 1.54%, respectively; Figure 4A). Further analysis revealed that 56.13% (87/155) of meropenem-resistant genomes harbored predicted truncation in OmpK35, with protein lengths ranging from 6 to 90% relative to the full-length sequence from the start codon. Conversely, predicted truncations were only 7.18% (14/195) in meropenem-susceptible genomes, with protein lengths ranging from 7 to 87%.

#### 3.5.2 Virulence gene distribution

Among the genomes analyzed, the hypermucoid-associated gene *rmpA2* was present in 15.48% (*n* = 24) of meropenem-resistant genomes compared with 5.64% of meropenem-susceptible genomes. The virulence score (0–5) to each genome is assigned based on the presence of more than half of the genes present in the gene clusters, such as aerobactin (abst), yersinabactin (ybst), and colibactin (cbst). Comparison between the meropenem-resistant and -susceptible genomes revealed that around 70% of the genomes were assigned the score 1–4, while the meropenem susceptible genomes accounted for 46% of the genomes. Out of the meropenem resistance genomes, approximately 52% of them harbored yersiniabactin gene clusters. In case of meropenem-susceptible genomes, there were 38% of genomes identified to possess yersiniabactin gene clusters. There were 4.10% of the meropenem-susceptible genomes harboring genes for the gene clusters, such asyersiniabactin, aerobactin, and colibactin, while no genomes with a phenotype resistant possessed all three gene clusters together (Figure 4B).

### 3.6 Pan-genome analysis

**Figure 5:**
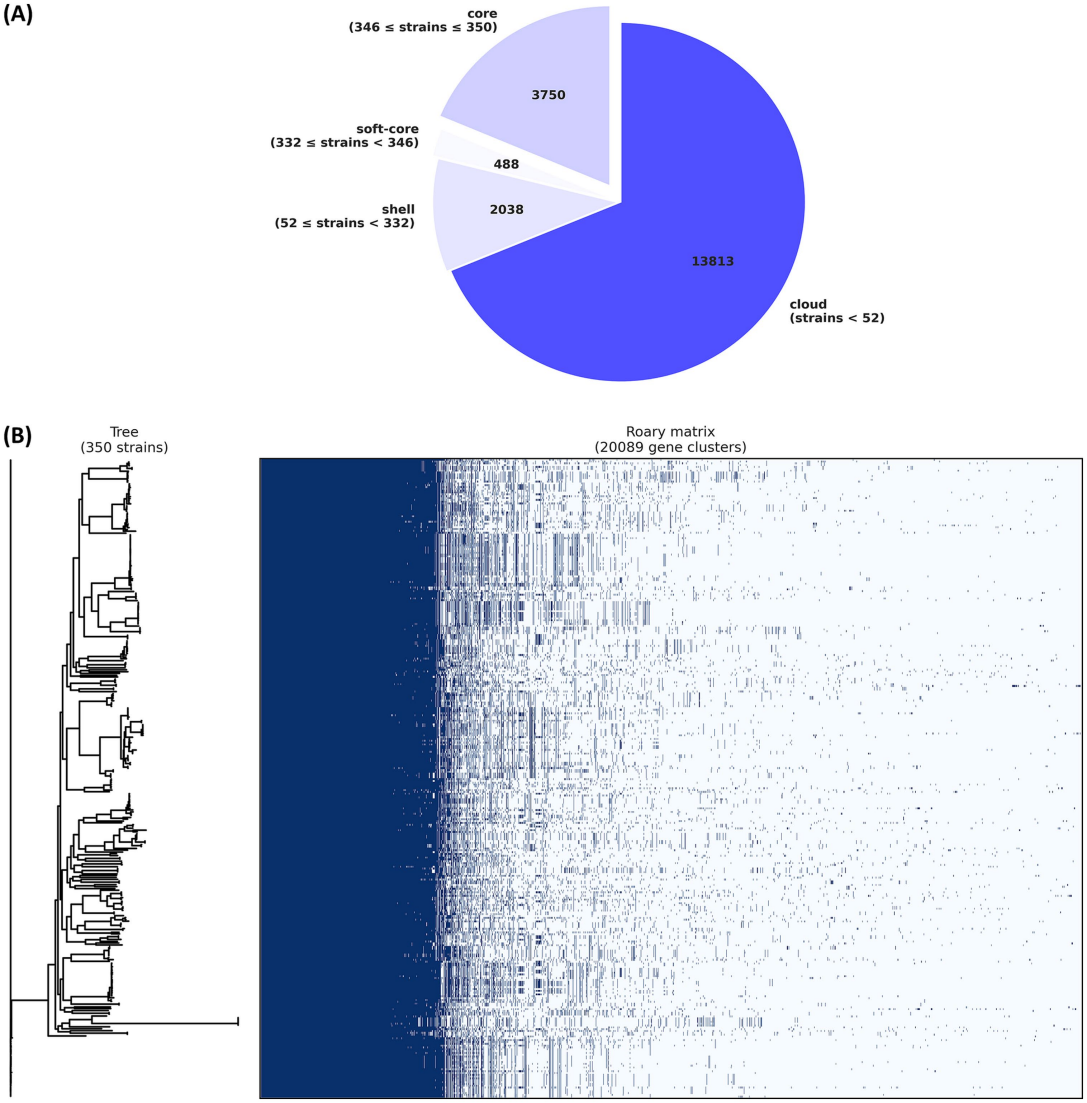
**(A)** Pie-chart representing the core and accessory genes across all *K. pneumoniae* genomes and **(B)** heatmap representing the presence–absence of genes among *K. pneumoniae* genomes.

The pan-genome analysis from Panaroo revealed 20,089 COGs, including 3,750 core genes present in 99–100% of genomes (346 ≤ strains ≤ 350), 488 soft-core genes present in 95–98% of genomes (332 ≤ strains < 346), 2,038 shell genes present in 15–94% of genomes (52 ≤ strains < 332), and 13,813 cloud genes present in < 15% of genomes (strains < 52). Core and soft-core genes were collectively considered the core genome, while shell and cloud genes were grouped as accessory genomes. Of the total genes, the core genome accounted for a smaller fraction of 21.1%, whereas the accessory genome, comprising shell and cloud genes, represented 78.90% (Figures 5A,B).

The core genome-based maximum-likelihood phylogeny showed that genomes predominantly clustered according to their STs. Similar clustering was also observed for K-locus and O-locus types. The resistance phenotype was predominantly associated with specific STs, namely ST101, ST11, and ST147. Furthermore, these resistance genomes were further populated in the clades in specific capsular types, particularly KL17 (*n* = 22), KL64 (*n* = 21), KL30 (*n* = 14), and KL24 (*n* = 12; Supplementary file 3 Figure S5).

**Figure 6:**
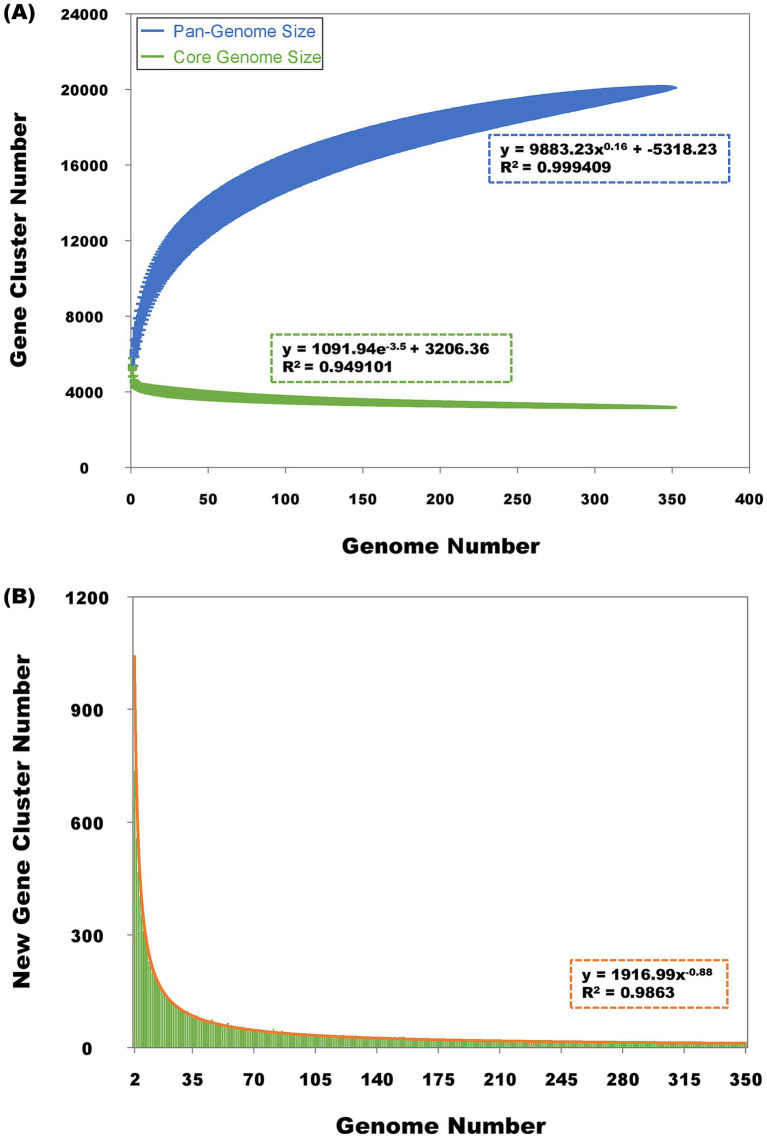
**(A)** The total number of core and pan-genome in the accumulation curve are represented in green and blue color. The pan-genome curve indicates open pan-genome structure as it increases upon the addition of new genomes **(B)** The bar graph represents the number of new gene clusters as *K. pneumoniae* genomes are added.

The gene presence–absence matrix was analyzed to understand the pan-genome structure. The pan-genome curve is represented by the model y = Ax^B^ + C, which showed a best-fit correlation of r^2^ = 0.999 (A = 9883.23, B = 0.16, and C = −5318.23). The value for Bpan of 0.16 represented the open pan-genome in *K. pneumoniae* genomes, indicating that as new genomes were added, the pan-genome size increases, while the core-genome decreases (Figure 6A). The new gene profile curve is represented by the function y = Ax^B^, with a correlation coefficient of r^2^ = 0.986 (A = 1916.99 and B = −0.88; Figure 6B).

Roary analysis revealed a total of 35,638 gene clusters with core genes comprising 3,282 genes, soft-core genes comprising 593 genes, shell genes comprising 2,161 genes, and cloud genes comprising 29,602 genes (Supplementary file 3 Figures S6A,B). The total genes in the accumulation curve illustrate the pan-genome’s expansion with the incorporation of additional genomes. This increase in total gene number and stable curve for conserved genes in the accumulation curve suggests the *K. pneumoniae* pan-genome is open (Supplementary file 3 Figure S6C). Furthermore, unique gene counts increased with the inclusion of more genomes, while the curve representing new genes exhibited a sharp and steady decline (Supplementary file 3 Figure S6D).

**Figure 7:**
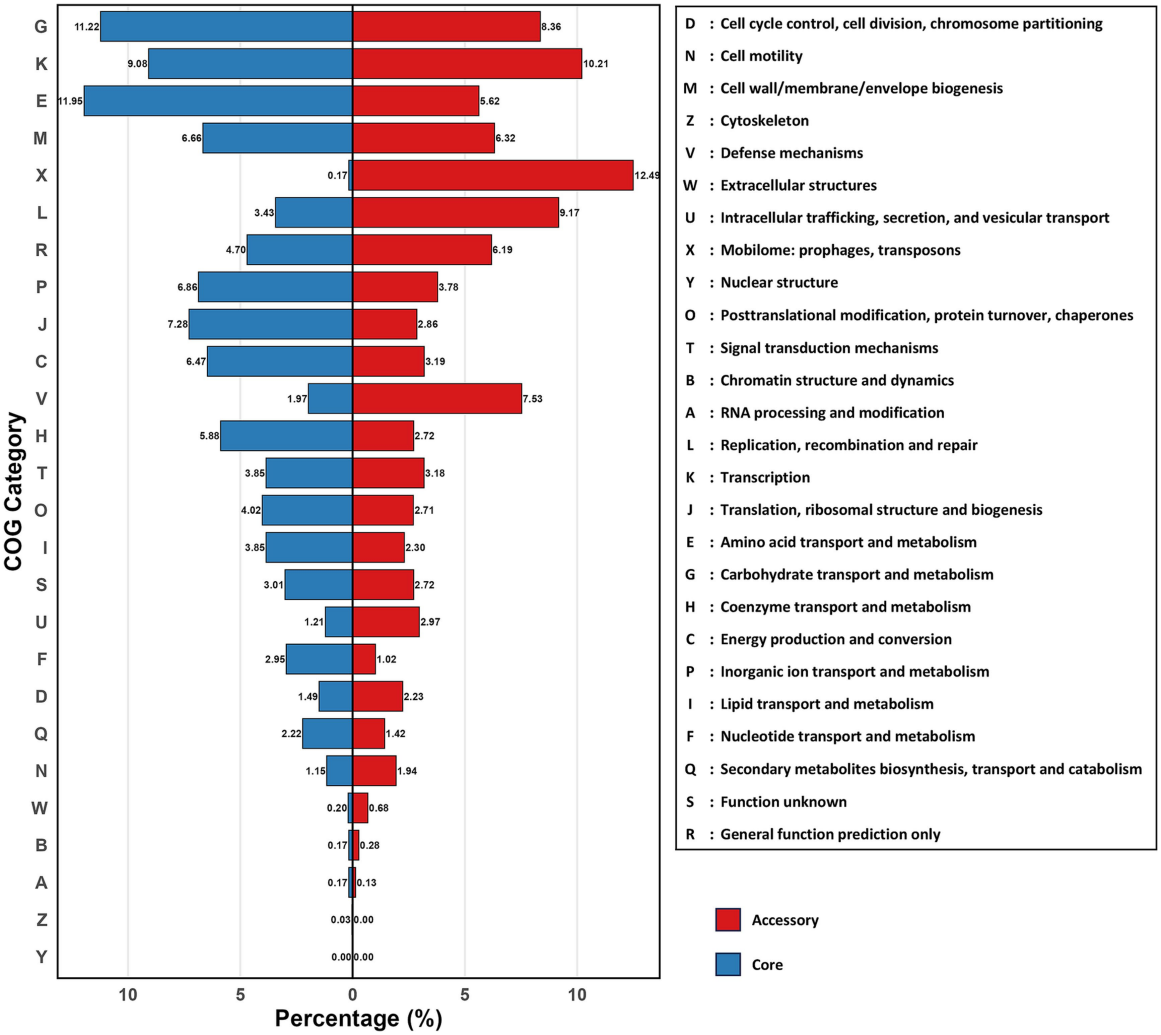
Comparison of percentage of genes classified for COG functional categories for core and accessory genes.

About 91.77% of the core genes were functionally classified for COG categories, while only about 54.82% was classified for accessory genes. The distribution of COG functional categories of genes belonging to core and accessory genes revealed that genes associated with metabolism were abundant in the core genes compared to accessory genes. The COG categories belonging to these metabolism related functions were energy production and conversion (COG C, 6.47%), amino acid transport and metabolism (COG E, 11.95%), nucleotide transport and metabolism (COG F, 2.95%), carbohydrate transport and metabolism (COG G, 11.22%), coenzyme transport and metabolism (COG H, 5.88%), lipid transport and metabolism (COG I, 3.85%), inorganic ion transport and metabolism (COG P, 6.86%), secondary metabolites biosynthesis, and transport and catabolism (COG Q, 2.22%). Genes associated with defense mechanisms (COG V) were approximately 3.8 times more prevalent in accessory genes than in core genes. Similarly, about 12.49% of the accessory genes were associated with COG functional category mobilome: prophages, transposons (COG X), which is approximately 73.47 times higher than the prevalence in core genes (0.17%; Figure 7).

The distribution of acquired ARGs was analyzed across the core and accessory genomes. No acquired ARGs were identified in the core genomes, whereas the accessory genome harbored a total of 854 ARG occurrences, representing 125 unique ARGs. Among these, 41 genes encoded β-lactamases from various classes. Particularly, class A β-lactamases identified in the accessory genome included *bla_CTX-M_* (5 variants), *bla_KPC_* (2 variants), *bla_SHV_* (11 variants), *bla_TEM_* (2 variants), *bla_LAP-2_*, *bla_SCO-1_*, *bla_VEB-5_*. Class B metallo β-lactamase comprised *bla_NDM_* (2 variants), *bla_IMP-18_*, *bla_VIM-1_*. Class C β-lactamase included *bla_CMY_* (4 variants) and *bla_DHA-1_*, while class D was represented by nine variants of *bla_OXA_*.

### 3.7 Mutation analysis

**Figure 8:**
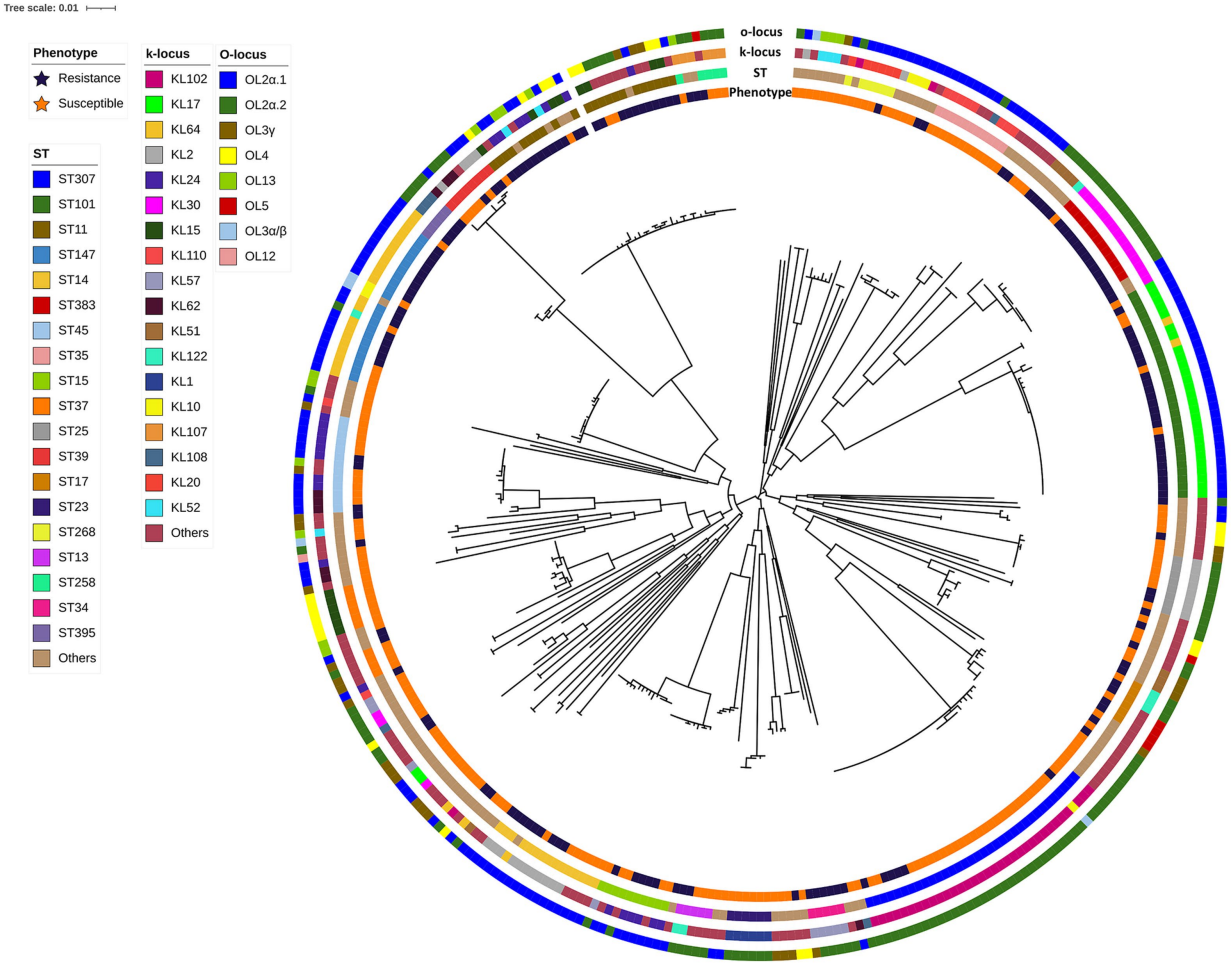
SNP-based phylogenetic tree with meropenem phenotype, ST, k-locus and o-locus annotations.

A total of 3,47,706 variants were identified, which belonged to SNPs (*n* = 2,98,905), MNPs (*n* = 41,814), indels (*n* = 5,320), and others (*n* = 1,667) across all genomes (*n* = 350). The recombination-free phylogeny constructed from core genome SNPs revealed clustering of the genomes that belonged to the same STs (Figure 8). A very common type of mutation in OmpK36 of meropenem-resistant strains was the insertion of glycine and aspartic acid between positions 135 and 136 (*n* = 39). There was a serine and aspartic acid insertion in OmpK36 in one strain of meropenem-resistant genome at the same position. Furthermore, insertion of aspartic acid and threonine between positions 136 and 137 was also found in nine strains of meropenem-resistant genomes, which was not in the susceptible strains. The aspartic acid insertion at position 136 in the OmpK36 protein was found in 11 meropenem-resistant strains. These insertions were not present or were very few in meropenem-susceptible genomes. The calculated pairwise SNP distance between the genomes ranged between 0 and 64,284, which was represented as a heatmap in Supplementary file 3 Figure S7. Visualization of the MSA of OmpK35 protein sequences clearly showed that more OmpK35 from meropenem-susceptible genomes were more intact compared to meropenem-resistant genomes (Supplementary file 3 Figures S8A,B).

A descriptive comparison of meropenem MIC distributions was analyzed separately according to CLSI and EUCAST criteria between genomes harboring carbapenemases alone and those with combined OmpK35 truncation, OmpK36 insertion, and carbapenemases (Supplementary file 5 Table S4A). Under EUCAST, genomes with combined mechanisms predominantly clustered within elevated MIC categories, particularly 16 mg/L (13 genomes) and >32 mg/L (20 genomes), whereas carbapenemase-only genomes showed a broader distribution, including ≥ 16 mg/L, 32 mg/L, >32 mg/L, and ≥128 mg/L categories. Detailed genome-wise resistance mechanism profiles used for MIC distribution analysis are presented in Supplementary file 5 Table S4B.

### 3.8 Genome-wide association study

GWAS analysis performed using a fixed effect model in Pyseer initially identified 167 genes/COGs significantly associated with meropenem phenotype. After adjustment for relevant covariates, including geographic location and collection year, the number of significant associations was reduced to 39 genes. Of these, 32 genes demonstrated a positive association with meropenem resistance. Among the positively associated genes, 13 genes encoded with known function, whereas 19 genes were classified as hypothetical proteins or not annotated with any known functions. The most significant associations were observed for *ble*, *trpF*, and *bla_NDM1_*. Additional genes significantly associated with meropenem resistance included *cutA_2*, *dsbD_3*/*nagA_2*, several *bla* variants, *intA_3*, *aacA4*, *repA_2*, *virB_1*, and *parA_1* (Supplementary file 6 Table S5A). Several of the significantly associated genes, including *ble*, *trpF*, *cutA_2*, *groL_2*, and *groS_2*, have previously been reported as components of the Tn125 transposon carrying *bla_NDM-1_*. Therefore, their association with meropenem resistance may reflect co-localization or genetic linkage with *bla_NDM-1_* rather than an independent functional contribution to carbapenem resistance. These genes may serve as genomic markers of resistance-associated mobile genetic elements, particularly Tn125-related structures.

A total of 619 SNPs were initially identified as significantly associated with the meropenem-resistance phenotype. However, after incorporating covariates to account for population structure and other confounding factors, only 12 SNPs remained significantly associated with phenotype. None of the missense variants showed a positive association with meropenem resistance, as all corresponding *β*-coefficients were < 0. Pyseer analysis performed with correction for population structure and covariates identified 54 unitigs significantly associated with the meropenem-resistance phenotype. Subsequent annotation of these unitigs to their corresponding regions mapped them to six unique genes, of which four were functionally characterized genes and two encoded proteins of unknown function (Supplementary file 6 Table S5B). The identified unitigs mapped to *yebV*, *hyfB*, *agp_1*, and *ppsR* showed significant effect sizes and were strongly associated with meropenem-resistant genomes.

The genetic markers that were associated with the meropenem phenotypes were assessed using Scoary identified that there were in total 53 genes that showed association with meropenem resistance. These identified gene clusters belonged to β-lactamases, such as metallo-β-lactamase (*bla_NDM-1_*) and oxacillinases (*bla_OXA-18_*), and were found to have a significant association with meropenem resistance. There were several transposases that were significantly associated with meropenem resistance, belonging to families such as IS1, IS4, IS5, IS91, IS1595, and Tn3. Among the meropenem resistance-associated genes, 20 gene clusters were not annotated to any known proteins and were labeled as “hypothetical protein.” Other genes that were identified were bleomycin-resistance gene (*ble*), multidrug efflux pump Tap, *trpF*, *cutA_2*, *groL_2/groS_2*, and *msr(E)*. The list of genes that were identified for association with meropenem resistance is provided in Supplementary file 6, Table S5C. About approximately 30% of genes (16/53) from the scoary results were associated with functions related to mobile genetic elements (MGEs), such as transposases belonging to insertion sequence, transposon-related proteins, and plasmid-associated proteins. This suggests that MGEs may facilitate the dissemination of ARGs.

## 4 Discussion

Meropenem is one of the important antibiotics used to treat difficult-to-treat infections caused by *K. pneumoniae*. However, the treatment efficacy of meropenem has been highly reduced due to improper or overuse of antibiotics, which facilitates the bacteria to evolve with various resistance mechanisms to escape from the antibiotic. In addition, the infections caused by CRKP are increasing, and the reduced efficacy of existing antibiotics, especially meropenem, is a great concern. Understanding the resistance mechanisms of these CRKP is an important strategy to preserve the antibiotic efficacy against the infections. The present study aimed at understanding the *K. pneumoniae* pan-genome and identification of genetic factors that contribute to resistance to meropenem through a genome-wide association study. Meropenem resistance mediated by porins, such as OmpK35 AND OmpK36, was also studied. Among the sequence types identified, meropenem-resistant genomes were predominantly associated with ST101, ST11, ST147, and ST383. Similarly, a previous study reported an increasing carbapenem resistance pattern among ST101 and ST383, where resistance to meropenem and imipenem increased from 33.33% in 2019 to 83.33% in 2021, indicating their clinical predominance (Gamaleldin et al., 2024). In addition, KL17 and KL64 were the most prevalent capsular loci among resistance genomes in this study. A previous study on MDR *K. pneumoniae* reported KL17, KL51, KL10, and KL64 as predominant capsular loci among major clones, indicating their prevalence (Huang et al., 2024). Capsular types mainly associated with virulence include K1 and K2, along with other types such as KN1, K57, K20, K5, K54, and K16 (Wang et al., 2025). In our study, KL2, which is associated with hvKP, was identified in 25 genomes belonging to multiple STs, such as ST14, ST25, ST380, ST39, and ST86, and carrying O-loci OL2α.1 and OL2α.2. Other capsular loci associated with hvKP identified in this study included KL57 (*n* = 9), KL20 (*n* = 6), and KL16 (*n* = 5).

There were diverse β-lactamases found across the genomes. Among the various β-lactamase classes, class A β-lactamases were the most abundant in genomes with both meropenem-susceptible and -resistant phenotypes. There were numerous variants for each of the class A β-lactamases (CTX-M, SHV, KPC, and TEM). The occurrence of a greater number of variants in β-lactamases is an indication of the mutations that occur in their wild-type structures. β-lactamases are one of the main resistance mechanisms against the antibiotic, and the mutations in them are an even greater challenge to address.

Carbapenemases, including KPC-1, NDM-1, and NDM-5, were more prevalent in meropenem-resistant genomes than in susceptible genomes. KPC-1 is a class A carbapenemase that hydrolyzes β-lactam antibiotics through an active-site serine residue, whereas NDM-1 and NDM-5 are class-B metallo-β-lactamases that require zinc ions for their catalytic activity. However, carbapenem resistance is often driven by the combined effect of multiple mechanisms rather than the presence of a single resistance determinant. Recent literature has reported that the co-occurrence of carbapenemases, such as *bla_KPC_* with metallo-β-lactamases (*bla_NDM_*, *bla_IMP_*, or *bla_VIM_*), dual metallo-β-lactamases (*bla_NDM_* - *bla_IMP_*), or *bla_OXA-48_* together with *bla_NDM-1_*, can enhance the complexity of AMR surveillance and treatment strategies (Yu et al., 2025). In addition, outer-membrane porin alterations and increased expression of efflux pumps can further contribute to carbapenem resistance in *K. pneumoniae* (Li et al., 2025).

The absence of detectable carbapenemase genes in 21 meropenem-resistant genomes in our study suggests the involvement of alternative resistance mechanisms. The presence of carbapenemase genes alone does not always correspond to phenotypic carbapenem resistance, as gene expression may be affected by promoter disruptions, low plasmid copy number, or other regulatory factors. For example, phenotypic carbapenem susceptibility, despite the presence of *bla_NDM-1,_* has been associated with deletion of the upstream promoter region, resulting in silenced gene expression (Qin et al., 2024). Carbapenem resistance in non-carbapenemase-producing strains may arise through reduced outer membrane permeability caused by porin alterations, overexpression of efflux pumps, or modifications in antibiotic targets such as penicillin-binding proteins (Lee et al., 2024). Prior literature demonstrates that carbapenem resistance within non-carbapenemase-producing *Enterobacterales* frequently arises from the co-occurrence of multiple acquired β-lactamases alongside porin deficiency mutations, underscoring the multifactorial etiology of this phenotype (Shortridge et al., 2022). There was a higher proportion of genomes that possessed more than three β-lactamases belonging to different classes. These findings underscore the importance of the effective therapeutic alternative for the β-lactamases inhibitors targeting a diverse range of β-lactamases.

It is a greater challenge in a clinical setting if the strains possess both antibiotic-resistant and hypervirulent characteristics, as it could cause severe infections, which could be difficult to treat (Russo and Marr, 2019; Shankar et al., 2021; Vijayakumar and Ramaiah, 2026). Based on the classification criteria implemented in Kleborate, genomes with a resistance score ≥1 and a virulence score ≥3 are considered convergent, indicating the co-occurrence of AMR and hypervirulence-associated features (Lam et al., 2021). In the present study, 29 of 155 (18.71%) meropenem-resistant genomes were identified as convergent strains belonging to several STs, including ST383, ST147, and ST231, suggesting the presence of resistance and virulence traits. Convergent CR-hvKP may arise either through the acquisition of AMR plasmids by hypervirulent strains or through the incorporation of virulence-associated plasmids into carbapenem-resistance lineages (Wang et al., 2025).

The distribution of genes among the COG functional categories for core and accessory genes revealed that genes corresponding to metabolism-associated functions were more predominant in the core genes, and the genes corresponding to mobilome and defence mechanism-related functions were higher in the accessory genes. These diverse genes related to mobilome and defence mechanism in the accessory genes suggest the possibility of these genes being disseminated to other strains of *K. pneumoniae* through horizontal gene transfer.

Further, the results from Kleborate revealed that around 56.13% of the meropenem-resistant genomes possessed possible truncated OmpK35 compared to meropenem-susceptible genomes (7.18%). Porins help in the entry of the antibiotics inside the bacterial cell, and any structural modifications in these porins will affect the efficacy of the antibiotics (Gogoi et al., 2023). Furthermore, the amino acid insertions of GD, TD, SD, and D were found in the OmpK36 protein of meropenem-resistant genomes, which were not present in the susceptible strains. Several studies have reported that modifications or loss of OmpK35 and OmpK36 in CRKP limit antibiotic entry across the outer membrane, thereby reducing the efficacy of several antibiotics, including carbapenems (Arabaghian et al., 2019; Wong et al., 2019; Veloso et al., 2023). A previous study found that 77.7% of CRKP isolates carried disrupted OmpK35, which was associated with increased carbapenem MICs, while concurrent loss of OmpK35 and OmpK36 further contributed to reduced carbapenem susceptibility (Bulman et al., 2021).

Interestingly, almost all meropenem-resistant genomes exhibited one or more resistance mechanisms in combination by possessing either possible truncated OmpK35 proteins or insertion in the OmpK36 proteins or harboring carbapenemases, such as *bla_IMP-18_*, *bla_KPC_* variants, *bla_NDM_* variants, *bla_OXA_* variants. Metallo β-lactamase, particularly NDM, representing transmissible carbapenemase, along with high prevalence of *ble_MBL_* among NDM-carrying strains, suggests a potential role in stabilizing the traits of NDM-related resistance (Dortet et al., 2012). The results from PySeer and Scoary-based GWAS in our study identified a significant association of *bla_NDM-1_*, *ble*, *trpF*, and *cutA_2* with meropenem resistance.

The gene composition of the Tn125 transposon carrying *bla_NDM-1_* comprises genes, namely insertion sequences, *ble*, *trpF*, *dsdC*, *cutA*, *groES*, *groEL*, and an incomplete TnAs3-Tn3 (Alghoribi et al., 2021). In the present study, most of these genes, such as *bla_NDM-1_*, *ble*, *trpF*, *cutA_2*, *groL_2*, and *groS_2,* were identified as significant determinants associated with meropenem resistance through GWAS analysis. This highlights the role of these genetic determinants, facilitating the dissemination of the resistance-associated genes. Furthermore, GWAS analysis identified the *rmtB* gene as significantly associated with meropenem resistance. This gene is associated with other resistance determinants, including ESBLs and carbapenemases, with a previous study in *Enterobacterales* reporting the chromosomal co-occurrence of *bla_NDM-5_* and *rmtB1* on chromosomes (Sabtcheva et al., 2024). Multiple transposases belonging to the IS1, IS4, IS5, IS91, IS1595, and Tn3 were significantly associated with meropenem resistance, suggesting that these mobile genetic elements may facilitate the horizontal dissemination of resistant genes. Several Tn125-associated genes, such as *ble*, *trpF*, and *cutA_2,* were significantly associated with meropenem resistance, supporting the role of Tn125-like mobile elements in the dissemination of *bla_NDM-1_*. Further validation is essential to determine the contribution of these genes to antibiotic resistance.

The genes *yebV*, *hyfB*, *agp_1*, and *ppsR* identified as statistically associated with meropenem resistance in our study have not been previously reported to directly contribute to meropenem resistance in *K. pneumoniae*. Among these genes, *hyfB* encodes formate hydrogenlyase subunit 3, whereas *agp_1* and *ppsR* encode glucose-1-phosphatase and phosphoenolpyruvate synthase regulatory kinase/phosphorylase, respectively, which are associated with metabolism-related processes. Although their role in AMR remains unknown, their statistical associations with meropenem resistance suggest that they may represent putative secondary resistance determinants or linked genetic markers. However, functional validations are required to determine whether these genes contribute mechanistically to meropenem resistance. Similar indirect associations of secondary resistance genes with carbapenem resistance have been reported in *Escherichia coli*, where *repA2* and *ccdB* were shown to support plasmid replication and maintenance under antibiotic stress (Baishya et al., 2021). Furthermore, the reduction in significant associations after adjusting for geographic location and collection year suggests that some initial associations may have been influenced by geographic and temporal confounding factors. Inclusion of these covariates reduced potential sampling bias and population structure effects, resulting in a set of genes more likely to be associated with meropenem resistance phenotype.

The key findings of this work expand our understanding of the genomic determinants contributing to meropenem resistance in clinically associated *K. pneumoniae* strains through integrated pan-genome analysis and genome-wide association study. Meropenem-resistant genomes were predominantly represented by ST101, ST11, ST147, and ST383, with KL17 and KL64 identified as the most prevalent capsular loci among these genomes. A wide range of β-lactamases were identified across the genomes, with class A enzymes widely distributed in both meropenem-resistant and susceptible genomes. In contrast, class B β-lactamases were particularly high among meropenem-resistant genomes. Notably, several resistant strains harbored multiple β-lactamase genes, underscoring their contribution to the resistance phenotype and the resulting therapeutic complications in managing *K. pneumoniae* infections. The functional annotation of core and accessory genomes based on COG classification revealed that essential metabolic processes were predominantly associated with the core genome, whereas mobilome and defense-related genes in the accessory genome emphasize their role in the horizontal dissemination of AMR determinants. Additionally, 52 genomes exclusively belonging to the meropenem-resistant phenotype co-harbored possible truncated OmpK35 and amino acid insertions in OmpK36, highlighting the contribution of outer membrane porin alterations to meropenem resistance. Genome-wide association analysis further identified several genes significantly associated with the resistance phenotype, including *blaNDM-1*, *ble*, *trpF*, *cutA*, *groL*, and *groS*. Overall, this study presents multiple resistance strategies, including harboring carbapenemases, porin modifications, and other MGEs, which highlight their relevance for genomic surveillance.

Taken together, this study highlights the value of genome-based approaches in deciphering the molecular basis of meropenem resistance in clinically relevant *K. pneumoniae*. However, the lack of corresponding virulence phenotypic data limited our ability to perform comprehensive genotype–phenotype association analyses for virulence-related determinants. In addition, binary phenotype classification may have limited the detection of subtle resistance-associated genetic variations. The uneven geographic distribution of publicly available genomes may have influenced the observed ST distributions and GWAS associations despite adjustment for geographic location and collection year. Future studies, such as larger and more geographically diverse genome datasets, together with further experimental validation, including resistance reconstruction, gene knockout, complementation, and MIC restoration assays, are required to strengthen the interpretation of genes associated with the resistance phenotype.

## 5 Conclusion

The findings of this study provide a comparative perspective on the determinants of meropenem resistance in *K. pneumoniae*. Diverse β-lactamases were detected in both meropenem-resistant and -susceptible genomes, with a notable predominance of carbapenemases, particularly, *bla_NDM_* and *bla_KPC,_* among the meropenem-resistant genomes. Additional resistance mechanisms included predicted truncations in the OmpK35 proteins, along with the insertional mutation (GD, TD, SD, and D) in OmpK36 proteins. The mobilome and the defense-related genes were dominated within the accessory genome, highlighting the potential of dissemination of AMR. Overall, these findings emphasize the importance of genomic surveillance and the need for the development of strategies to overcome the threat posed by CRKP.

## Data Availability

The original contributions presented in the study are included in the article/Supplementary material, and further inquiries can be directed to the corresponding author.
