# Supplementary material for: A comparative genomic approach to identify determinants of meropenem resistance in Klebsiella pneumoniae using pan-genome-wide association analysis
Source: Front Microbiol. 2026 Jun 19;17:1851170. doi: 10.3389/fmicb.2026.1851170 (PMC13328277; doi:10.3389/fmicb.2026.1851170)
Supplement: Supplementary file 1 [file Data_Sheet_1.PDF]

Supplementary Figures

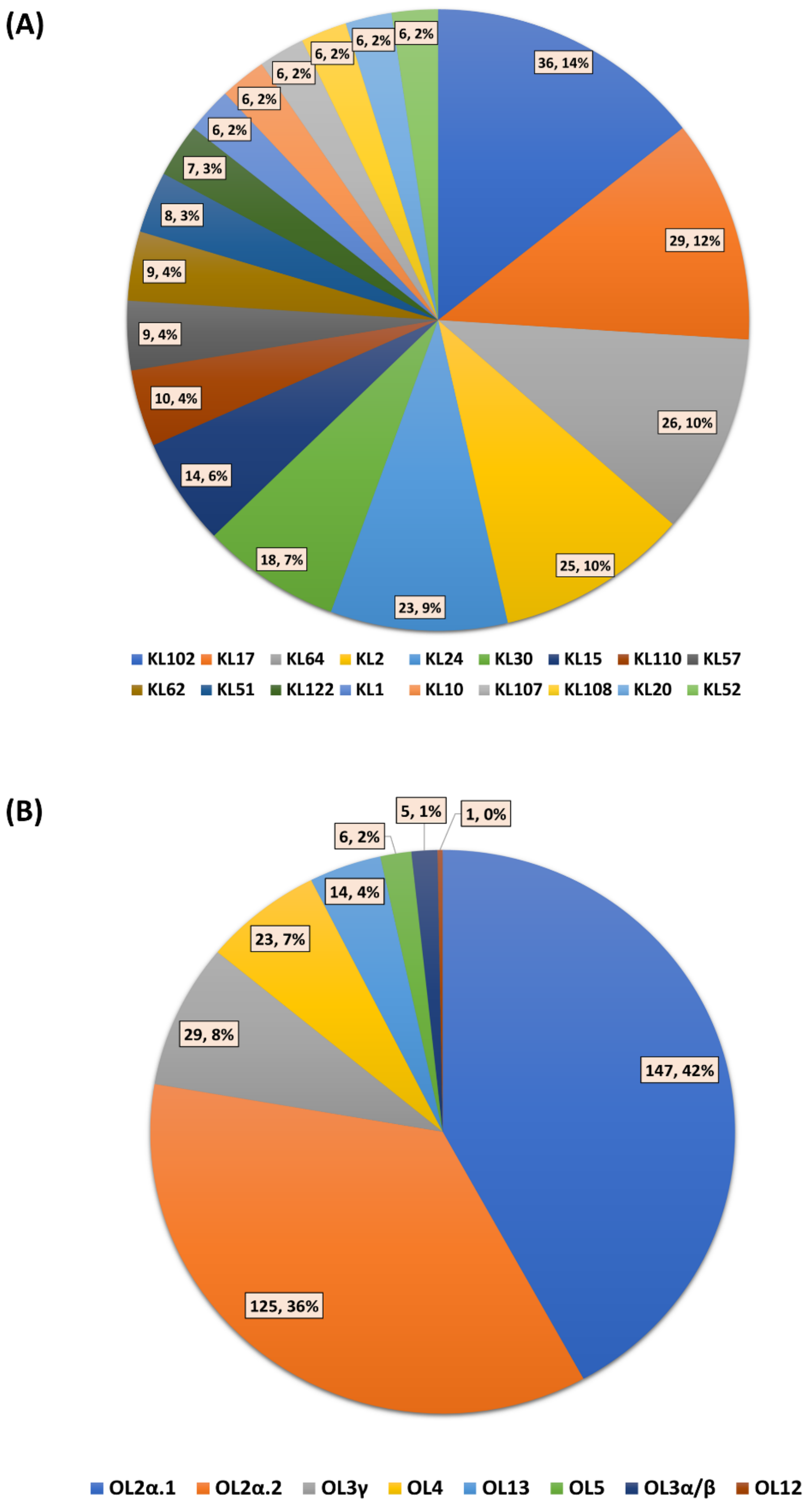

Figure S1. (A) Distribution of K-locus types present in more than five *K. pneumoniae* genomes (B) Distribution of O-locus types across all *K. pneumoniae* genomes

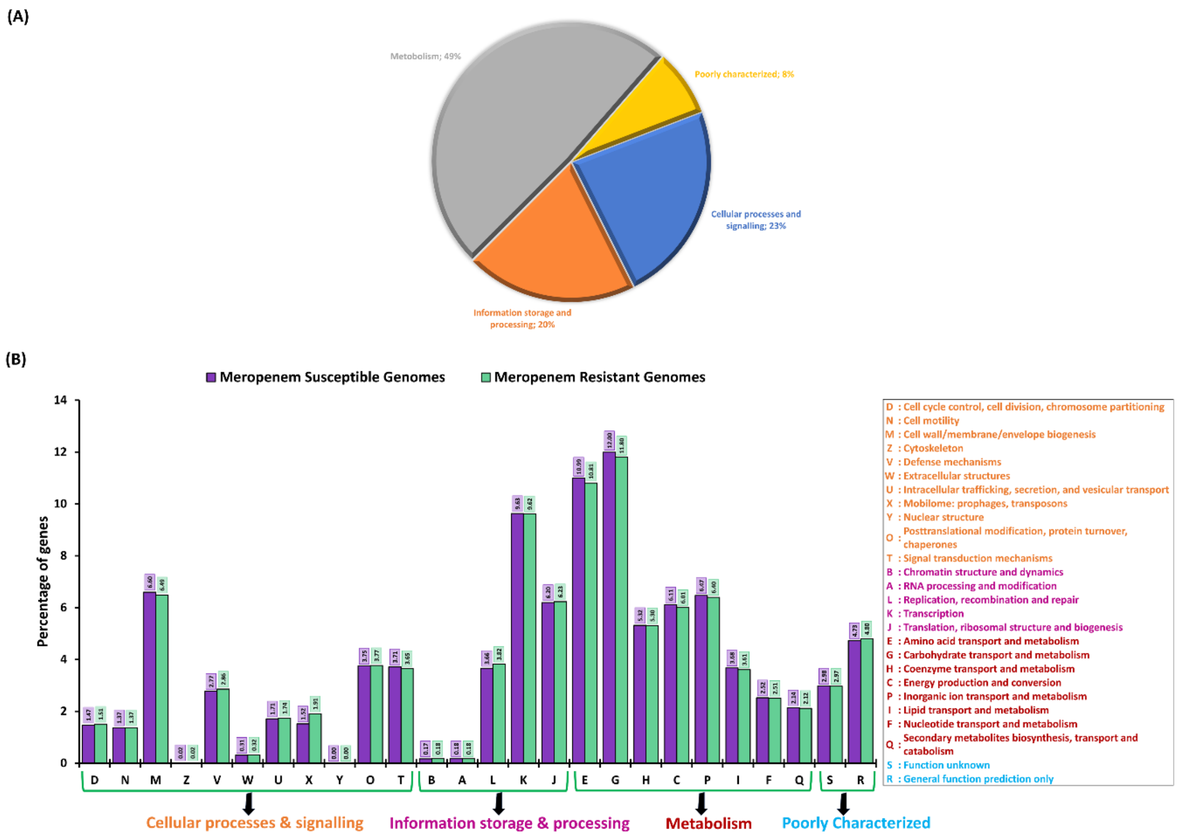

Figure S2. (A) Distribution of genes across the 4 major groups (B) Comparison of average gene counts between meropenem resistant and susceptible *K. pneumoniae* genomes

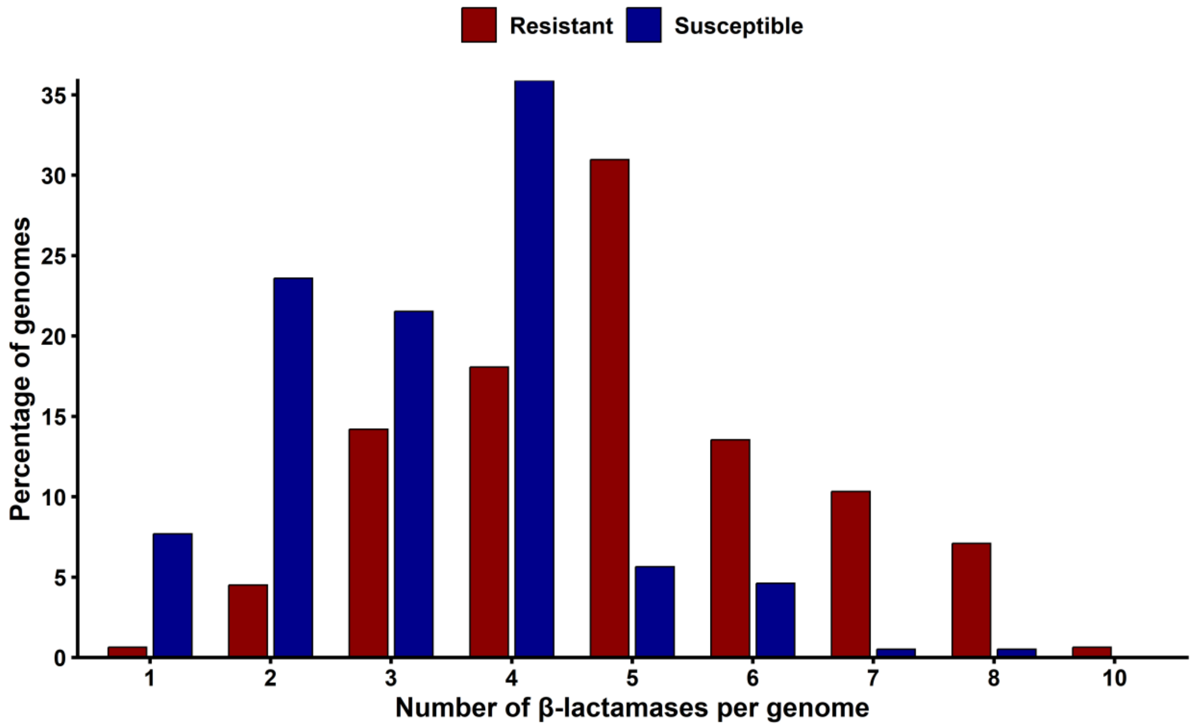

Figure S3. Distribution of β-lactamases per genome for meropenem-resistant and susceptible *K. pneumoniae* genomes

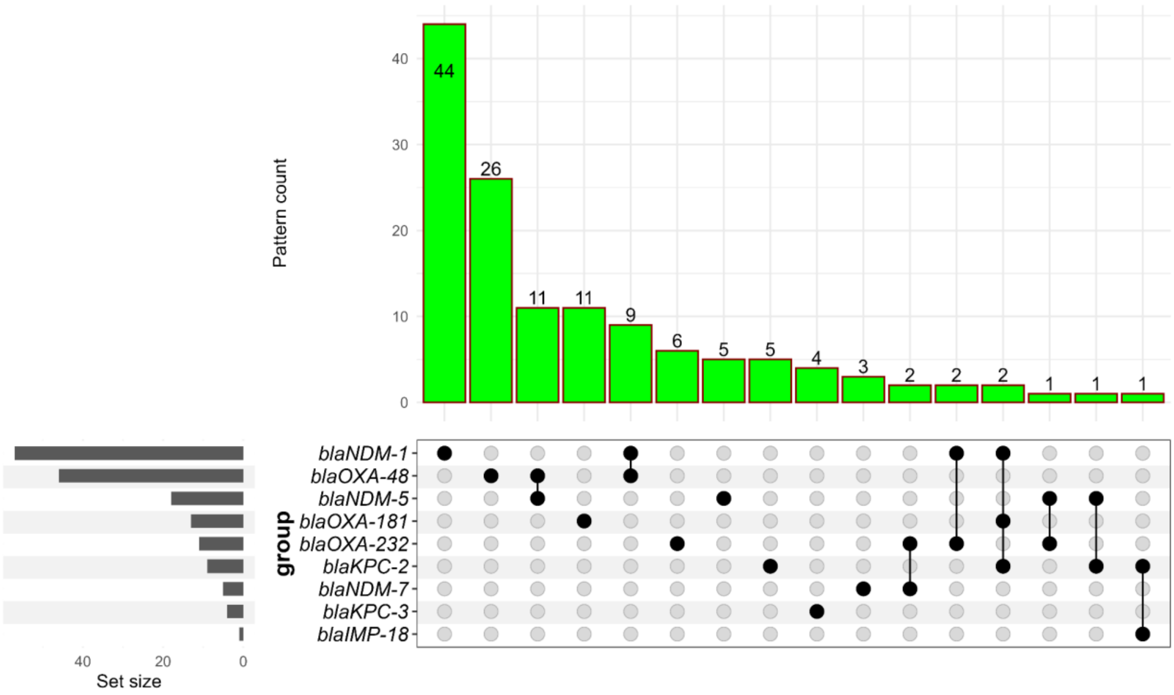

Figure S4. Upset plot representing distribution of carbapenemase gene combinations among meropenem-resistant *K. pneumoniae* genomes

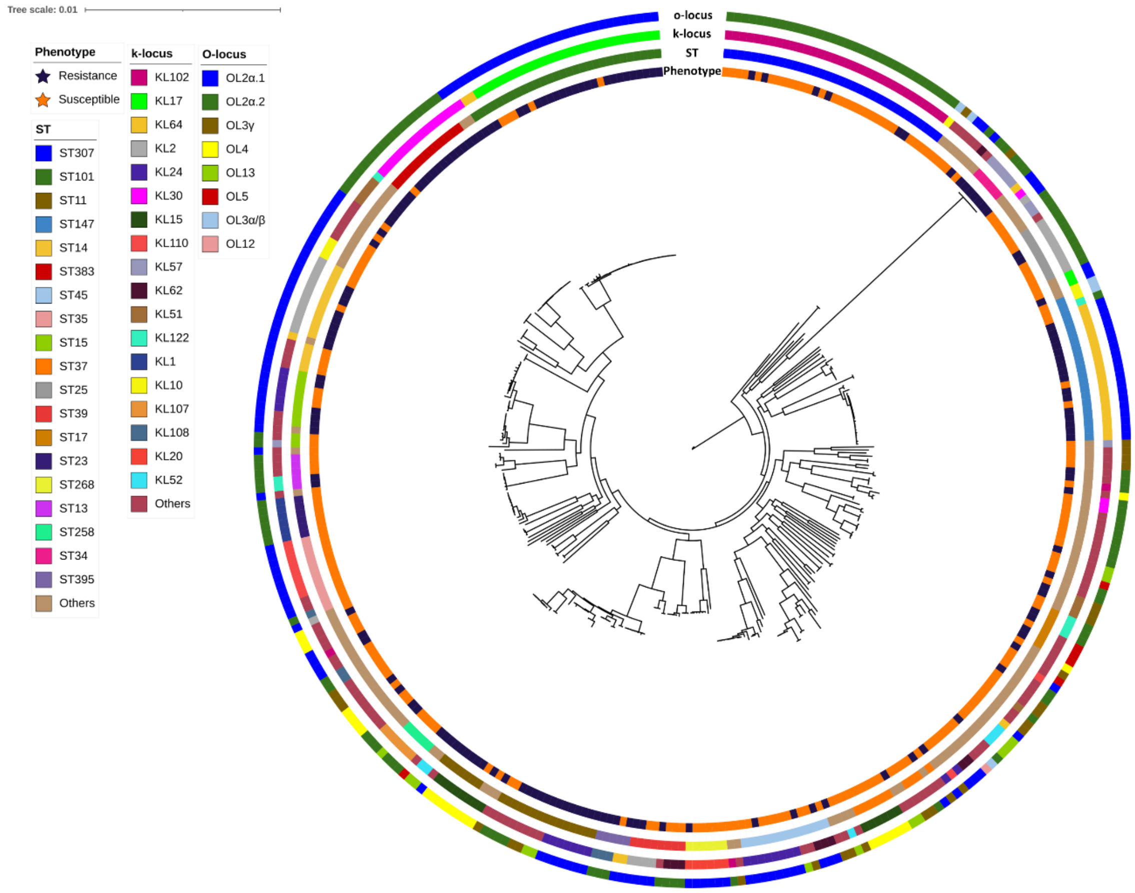

Figure S5. Core-genome based phylogeny annotated with meropenem susceptibility phenotype, STs, k-locus, and o-locus from inner to outer rings of the phylogeny

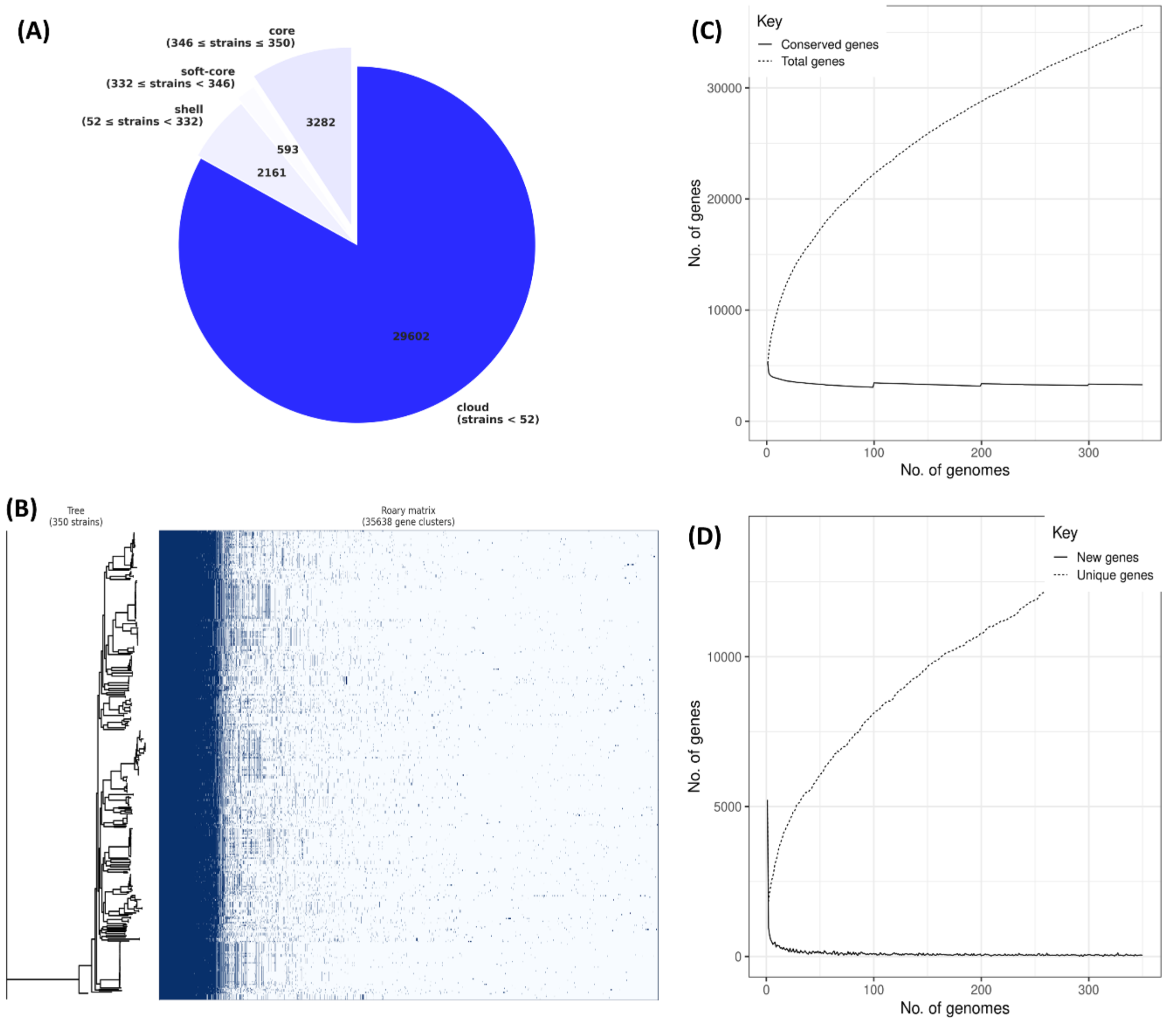

Figure S6. Results of pan-genome analysis from Roary (A) Pie-chart represents the distribution of the core and accessory genes (B) Heatmap representing the presence and absence of gene clusters (C) Plot representing the distribution of the total and conserved genes among the *K. pneumoniae* genomes (D) Plot representing the new and unique gene distribution among *K. pneumoniae* genomes

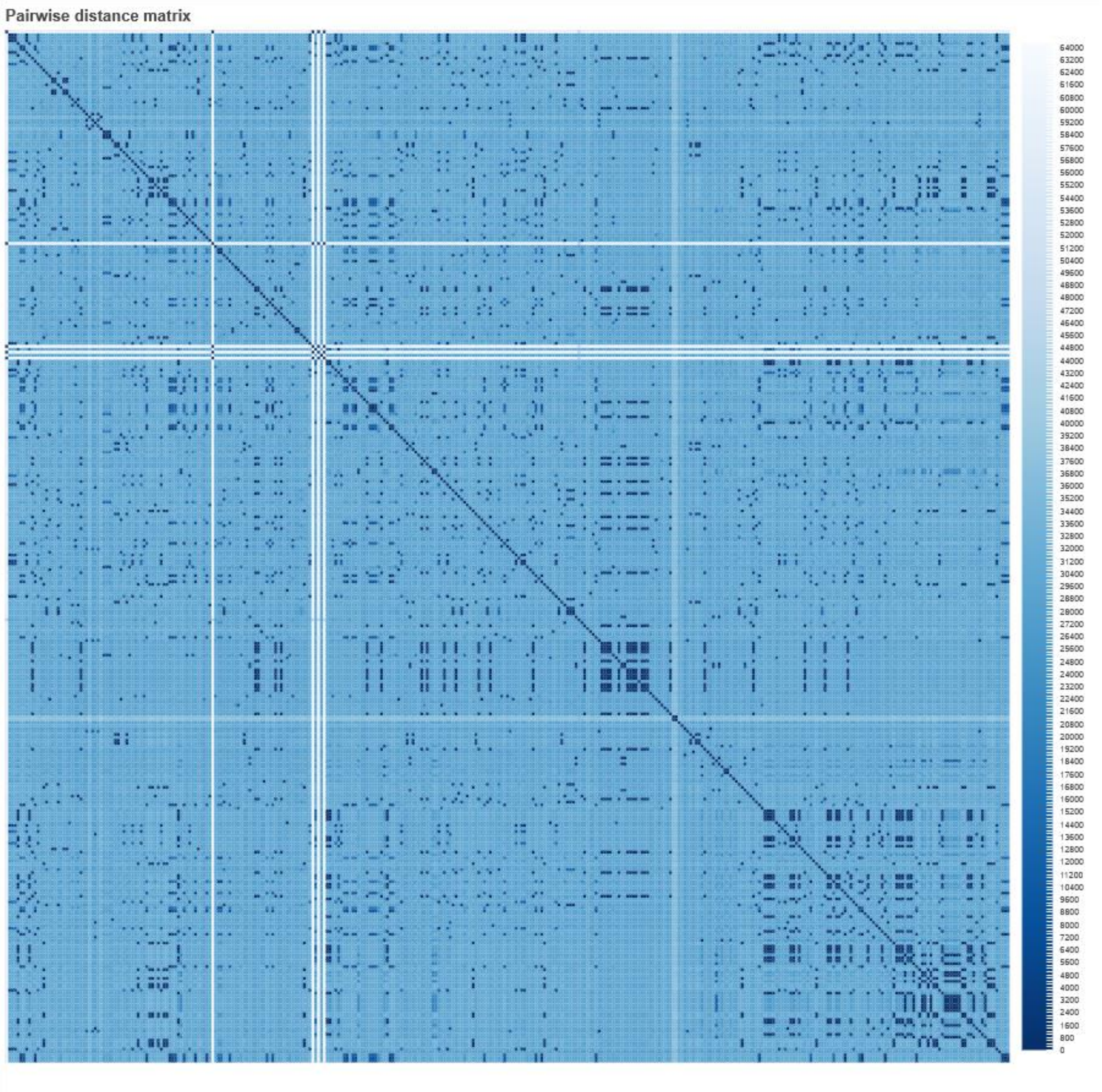

Figure S7. Heatmap representation of the pairwise SNP distance between *K. pneumoniae* genomes

(A)

(B)

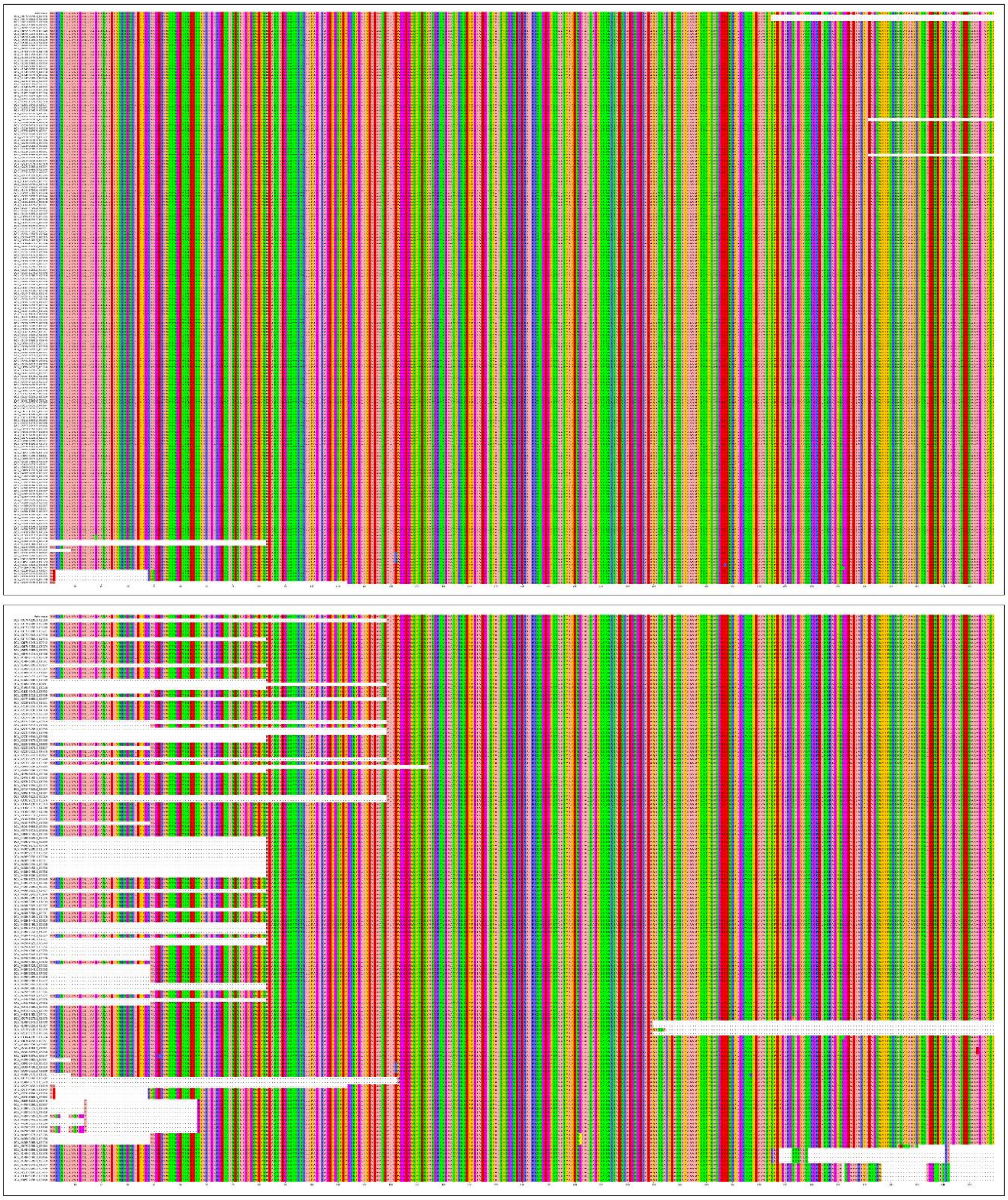

Figure S8. Visualization of MSA of OmpK35 protein (A) Alignment of OmpK35 protein sequences of meropenem-susceptible genomes (B) Alignment of OmpK35 protein sequences of meropenem-resistant genomes
